# Antimalarial Iron Chelator, FBS0701, Shows Asexual and Gametocyte *Plasmodium falciparum* Activity and Single Oral Dose Cure in a Murine Malaria Model

**DOI:** 10.1371/journal.pone.0037171

**Published:** 2012-05-21

**Authors:** Patricia Ferrer, Abhai K. Tripathi, Martha A. Clark, Carla Cerami Hand, Hugh Young Rienhoff, David J. Sullivan

**Affiliations:** 1 The Malaria Research Institute, W. Harry Feinstone Department of Molecular Microbiology and Immunology, The Johns Hopkins Bloomberg School of Public Health, Baltimore, Maryland, United States of America; 2 Department of Microbiology and Immunology, University of North Carolina, Chapel Hill, North Carolina, United States of America; 3 Department of Epidemiology, School of Public Health, University of North Carolina Chapel Hill, North Carolina, United States of America; 4 FerroKin BioSciences, Inc., San Carlos, California, United States of America; Université Pierre et Marie Curie, France

## Abstract

Iron chelators for the treatment of malaria have proven therapeutic activity *in vitro* and *in vivo* in both humans and mice, but their clinical use is limited by the unsuitable absorption and pharmacokinetic properties of the few available iron chelators. FBS0701, **(S)3”-(HO)-desazadesferrithiocin-polyether [DADFT-PE],** is an oral iron chelator currently in Phase 2 human studies for the treatment of transfusional iron overload. The drug has very favorable absorption and pharmacokinetic properties allowing for once-daily use to deplete circulating free iron with human plasma concentrations in the high µM range. Here we show that FBS0701 has inhibition concentration 50% (IC_50_) of 6 µM for *Plasmodium falciparum* in contrast to the IC_50_ for deferiprone and deferoxamine at 15 and 30 µM respectively. In combination, FBS0701 interfered with artemisinin parasite inhibition and was additive with chloroquine or quinine parasite inhibition. FBS0701 killed early stage *P. falciparum* gametocytes. In the *P. berghei* Thompson suppression test, a single dose of 100 mg/kg reduced day three parasitemia and prolonged survival, but did not cure mice. Treatment with a single oral dose of 100 mg/kg one day after infection with 10 million lethal *P. yoelii* 17XL cured all the mice. Pretreatment of mice with a single oral dose of FBS0701 seven days or one day before resulted in the cure of some mice. Plasma exposures and other pharmacokinetics parameters in mice of the 100 mg/kg dose are similar to a 3 mg/kg dose in humans. In conclusion, FBS0701 demonstrates a single oral dose cure of the lethal *P. yoelii* model. Significantly, this effect persists after the chelator has cleared from plasma. FBS0701 was demonstrated to remove labile iron from erythrocytes as well as enter erythrocytes to chelate iron. FBS0701 may find clinically utility as monotherapy, a malarial prophylactic or, more likely, in combination with other antimalarials.

## Introduction

Iron metabolism is a proven target for many malaria drugs. The quinolines like chloroquine and quinine interfere with iron protoporphyrin IX crystallization in the digestive vacuole [1]. The artemisinins are activated by iron to generate carbon-centered radicals that rapidly kill parasites [2]. Iron chelators have been explored as alternative malaria drugs for decades [3]. Intravenous deferoxamine does increase clearance of parasites [4] but in an important clinical trial deferoxime did not effect outcome from severe cerebral malaria [5]. The orally available deferiprone was also suboptimum in an uncomplicated malaria trial because of poor iron clearance after oral absorption [6]. Iron regulation is potent in limiting liver stage multiplication mediated, in part, by hepcidin pathway [7]. Iron supplementation increases hepatic parasites [8] and iron chelators decrease hepatic parasites [7]. For intraerythrocyte *Plasmodium,* the literature describes chelation of both labile iron pools in parasites as well as infected erythrocyte cytosol. Deferoxamine decreases available iron in the nucleus and parasite cytosol. The exact mechanism of parasite killing is not known but evidence points to a iron chelator mechanism of limiting iron for ribonucleotide reductase production of nucleotides, however earlier studies also suggest a toxic mechanism of erythrocytic *Plasmodium* killing [3]. A drawback for *Plasmodium* iron chelation chemotherapy is interference with the parasiticidal action of artemisinin drugs [9], [10].

The potent novel chemical analogue of the bacterial siderophore desferrithiocin is entering clinical trials for transfusional iron overload. **(S)-4,5-dihydro-2-[2-hydroxy-3-(3,6,9-trioxadecyloxy)phenyl]4-methyl-4-thiazolecarboxylic acid** also known as (S)3”-(HO)-desazadesferrithiocin-polyether [DADFT-PE] or more simply as FBS0701 has a very high affinity and specificity for Fe (III) with an equilibrium constant of 10^−22^ Molar [11]. The molecular weight of the formulated salt is 441 while free base is 400. The iron clearing efficiency in rodents is 10.6+4.4% and in primates is 23+4.1% [11], [12]. In contrast the iron clearing efficiency for deferoxamine is 2.8% and 5.5% and deferiprone is 1.2% and 2.1% in rodents and monkeys, respectively. In normal human volunteers dosed with FBS0701 with 6, 10 or 16 mg/kg, the pharmacokinetics across the dosing range indicates high bioavailability C_max_ of 22.7, 41.5 and 87.0 µM respectively, dose-proportionality and a terminal t_1/2_ permitting once-daily dosing [13].

Here we investigated the actions of this iron chelator on *P. falciparum* blood stage parasites, its action against gametocytes and efficacy in murine malaria models.

## Methods

### Materials

FBS0701 was obtained from Aptuit (Kansas City) LLC under good manufacturing practice. All other reagents and chemicals are from Sigma unless otherwise noted. *P. falciparum* isolates 3D7, Indo and KMVII were obtained from ATCC-BEI MR4 repository.

### *P. falciparum* culture

Direct effect of FBS7010 on *P. falciparum* was determined by SYBR green I (Invitrogen-Molecular Probes, Eugene OR) based assay as described earlier [14]. Briefly, the inhibition assay was initiated by adding serially diluted FBS7010 in 96 well microplates followed by sorbitol synchronized 1% ring stage parasites at 1% hematocrit in RPMI 1640 media with glutamine and HEPES supplemented with 10% human serum and 50 mg/ml hypoxanthine. Culture plates were then transferred to 37°C incubator and maintained in the environment of 5% O_2_, 5% CO_2_ and 90% N_2_ for 72 hrs. 2× SYBR Green I solution in lysis buffer was added after one cycle of freeze and thaw. Plates were then placed at room temperature in dark for 1–2 hours and fluorescence values were quantitated in a plate reader (HTS 7000, Perkin Elmer) at emission and excitation wavelengths of 535 and 485, respectively. For fractional inhibition analysis quinine, quinidine, chloroquine and artemisinin were used at concentrations ranging from 1 to 100 nM and FBS0701 from 1–15 uM. The fractional inhibition was analyzed according to the original Elion and Hitchings paper [15] with modifications by Bell [16]. The fraction of the chloroquine, quinine or artemisinin drug in combination with FBS0701 that produced an IC_50_ was divided by the IC_50_ of the chloroquine, quinine or artemisinin alone for the ratio on the y-axis. The x-axis was fraction of FBS0701 in combination with the chloroquine, quinine or artemisinin divided by concentration of FBS0701 which produced an IC_50_.

To test the effect of FBS0701 on gametocyte stages, *P. falciparum* NF54 cultures were initiated in 24 well plates at 0.5% asexual parasitemia and 4% hematocrit [17]. Medium was changed daily up to day 18, without addition of fresh RBCs. Continuous cultivation without dilution leads to concomitant crash of asexual parasitemia and induction of gamteocytogenesis by day 5. The gametocytemia was approximately 7% at start of treatment. FBS0701 was dosed in wells at Days 9–10 , when majority of gametocytes were in early stage of development (Stage I, II) or Days 14–15 when majority of gametocytes have matured to stage III to V. Levels of gametocytemia were determined on day 18 and the mean number of gametocytes was calculated by counting 10 high-powered (1000×) fields from triplicate wells per condition. More than 1000 erythrocytes were enumerated by random scanning across Giemsa blood film.

### Animal Ethics

All animal experiments were performed on protocol “Malaria Drug Testing in Mice” (Approval ID-MO09H401) approved by The Johns Hopkins Animal Care and Use Committee in accordance with institutional standards.

### Malaria Murine Drug Testing

Male 5–6 week old at approximately 25±2 g C57/BL6 mice were purchased from Jackson Laboratories (Maine, USA). The *P. berghei* ANKA strain was obtained from ATCC; the *P. yoelii* 17× lethal strain was the gift of James Burns (Drexel University). Freshly made solutions of FBS0701 in water were used for all the experiments and drug was administered by oral cannulation at indicated times. Murine infection was initiated by intraperitoneal inoculation of a million parasites for *P. berghei* and 10 million for *P. yoelii*. Blood was taken from the tail vein for blood smears. Parasitemias were determined in a blinded fashion by counting four fields of approximately 200 erythrocytes per field. Mice that survived for 30 days post infection with complete disappearance of parasitemia and no recrudescence within the next 30 days were considered cured.

### Pharmacokinetics

30 Balb/c mice were dosed with 100 mg/kg of FBS0701 by oral cannulation and blood was drawn by cardiac puncture during anesthesia. Whole blood was separated into plasma and erythrocytes and plasma sent for analysis by Covance Laboratories (Madison, WI) using a validated assay [13].

### Measurement of Bioavailable Labile Iron in Erythrocytes

Blood was collected from healthy subjects washed 2× with PBS. Erythrocytes were then incubated with 0.125 uM calcein (Invitrogen) in PBS for 15 minutes at 37C, washed 2× with PBS and then allowed to rest for 10 minutes at 37C in the dark [18], [19], [20]. Cells were incubated with PBS, 100 µM FBS0701 or 100 µM diferiprone for 1 hour at 37C. Cells were analyzed by flow cytometry using a modified FACS-Calibur with 2 lasers 30 mW 488 Diode Pumped Solid State laser and a 25 mW 637 red diode laser (FACS-Calibur; Becton Dickinson, Mount View CA, modified by Cytek Development). Data was collected using FlowJo CE and analyzed using Summit v4.3.01. The emission of 400,000 cells was analyzed using logarithmic amplification for fluorescence signal height (FL-1, 530/30) and linear amplification for forward scatter (FSC) and side scatter (SSC). The threshold was set at FSC to exclude cell debris and microparticles. The mean fluorescence intensity (MFI) was calculated using Summit v4.3.01. Individual experiments were performed in triplicate and also biologic duplicate with different human erythrocyte donors. The statistical significance was calculated using the Student’s t-test.

## Results

### *P. falciparum* Blood Stage Inhibition

**Figure 1:**
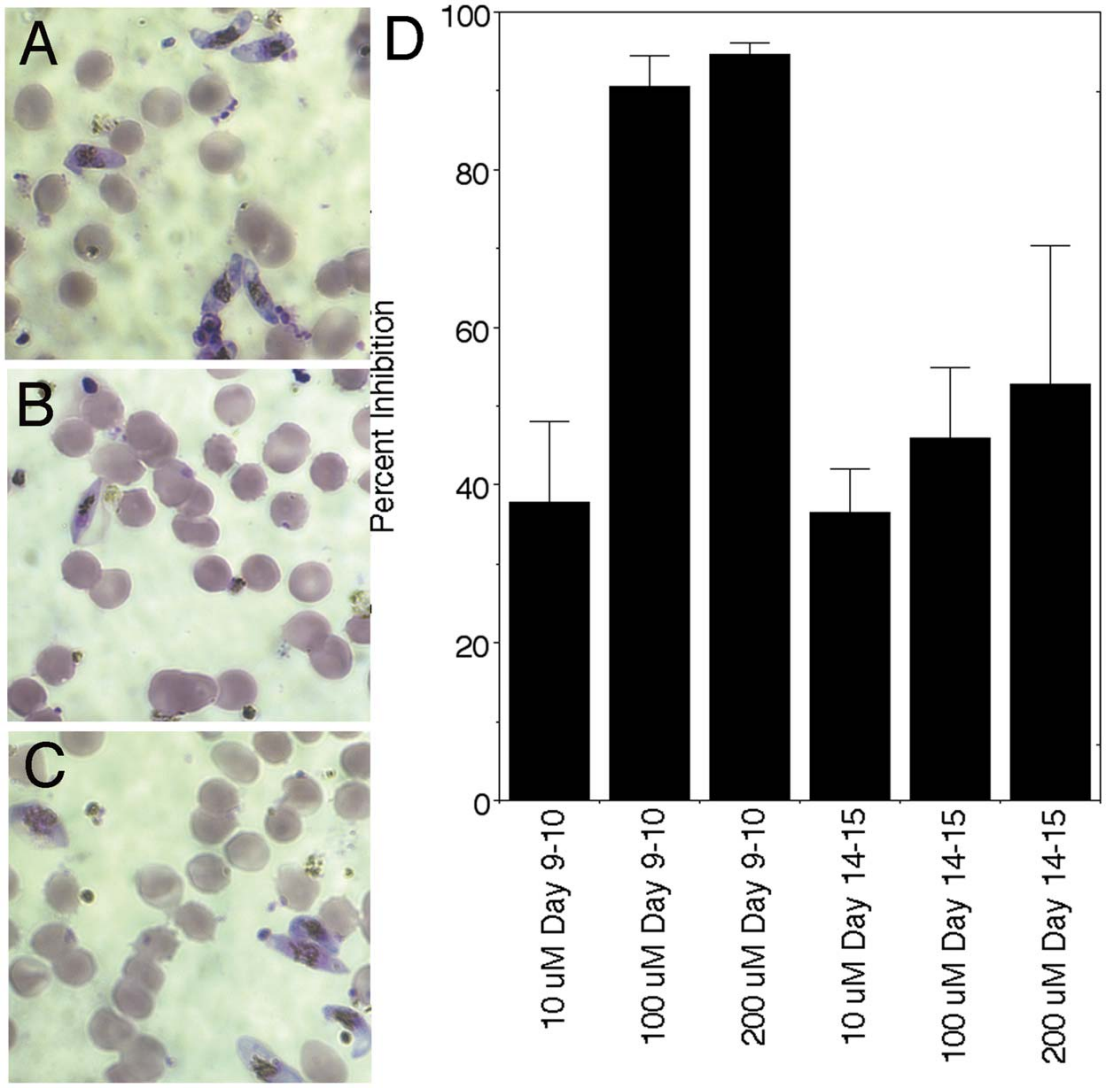
FBS0701 inhibits early stage not late stage gametocytes. Gametocyte cultures of *P. falciparum* NF54 strain were initiated at 0.5% asexual parasitemia and 4% hematocrit in 24 well culture plates. Gametocytes were visualized and counted on Day 18 with no drug (A) or 100 uM FBS0701 dosed in wells at Days 9–10 (B) or Days 14–15 (C) post-culture initiation. Dose dependent inhibition is more pronounced with early gametocyte stage I and II (day 9–10 dosing) than gametocyte late stages III-IV (day 14–15 dosing) (D). Gametocytes were inoculated at 0.5% parasitemia, resulting in gametocyte induction, followed by dosing of FBS0701 on indicated days. Gametocytes per 10 high power fields (excess of 1000 erythrocytes) in each of three replicate wells for each condition were counted on Day 18. Control wells had 7.4% mean gametocytes. Data expressed as percent inhibition of control. Standard deviation of triplicate observations for each of the three wells is shown.

**Figure 2:**
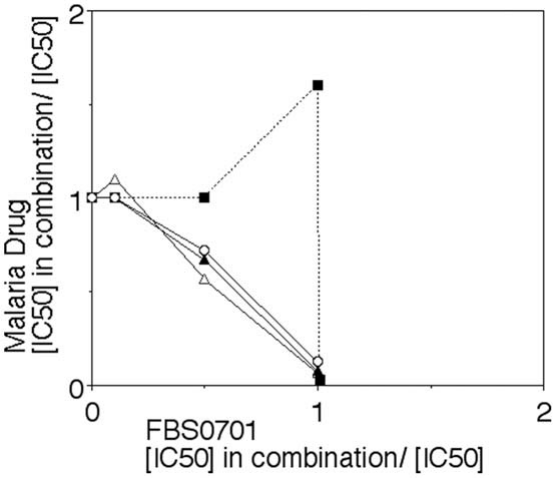
FBS0701 interferes with artemisinin but not the quinolines like chloroquine or quinine. Fractional inhibition curve for the quinolines and artemisinin with FBS0701. Up to 10 uM FBS0701 does not change the low nM IC50 of artemisinin (filled square). In contrast for the quinolines 1, 5, 7.5 and 10 uM FBS0701 reduced the IC50 in an additive manner for chloroquine (empty circle), quinine (empty triangle) or quinidine (filled triangle). For each axis the concentration of drug in combination which equals the inhibition concentration 50% was divided by IC50 of drug alone.

In the chloroquine and quinine sensitive isolate 3D7, the IC50 with continuous drug exposure was 6 µM (IC5 = 3 µM and IC90 = 10 µM). Testing in the chloroquine and quinine resistant parasites Indo and KMVII showed a similar inhibition to 3D7. Morphologically the FBS0701 exposed parasites were shrunken. Stage and concentration dependence of FBS0701 iron chelation showed that for continuous drug exposure starting at ring and trophozoite stage the IC_50_ was similar at 6 µM, but when drug exposure was initiated at schizont stage after DNA replication, the IC_50_ increased by almost three fold to 15–17 µM. Investigation of dose-dependent killing of gametocytes shows that 100 µM inhibited more than 90% of early stage I and II gametocytes but only about 40% of late stage III or IV gametocytes, 10 µM FBS0701 had minimal 37% decrease in the number of gametocytes (Figure 1). Because of the known interactions of iron chelators with the artemisinins [9], [16], [21], we investigated interaction *in vitro* with *P. falciparum*. Fractional inhibition indicates interference with artemisinin and an additive action with the quinolines like chloroquine or quinine (Figure 2).

### Murine Malaria Inhibition

**Figure 3:**
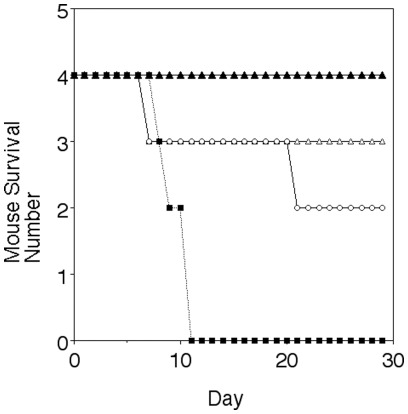
FBS0701 cures lethal *P. yoelii* in a single dose. Mice in groups of 4 were inoculated with 10 million lethal *P. yoelii* parasites by IP injection on day 0. A single oral dose of FBS0701 at 100 mg/kg given on day −7, day −1 and day +1 relative to infection. The survival curve shows complete protection with day +1 dosing and significant protection in animals pre-treated day −7 and day −1.

**Figure 4:**
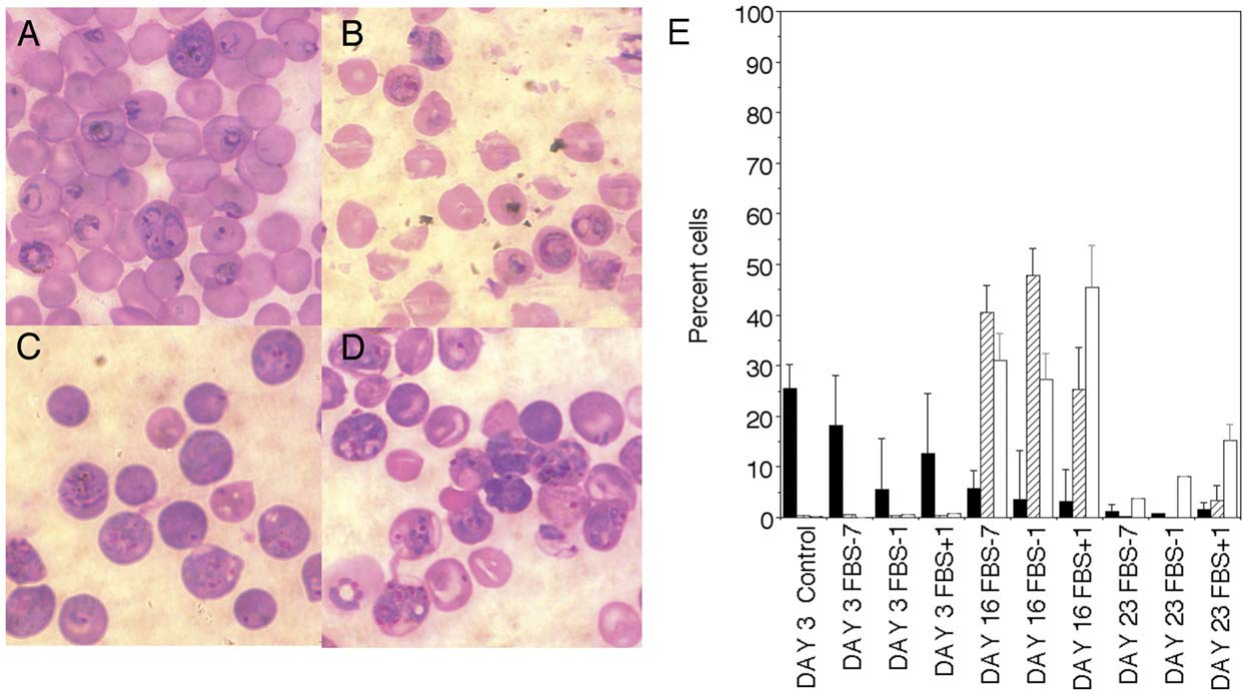
Protection is associated with exclusion of infection from normocytes. On day 3 blood films from control (A) and FBS0701 (B) treated mice both show infection in normocytes. On day 16 *P. yoelii* infection is largely restricted to young reticulocytes with only a few in normocytes in mice treated on day −7 (C) or day −1 (D). Quantification of blood films (E) indicated that on day 3 infected normocytes (black bars) out numbered by more than 20 fold infected reticulocytes (hatched bar). On day 16 most of the erythrocytes are either infected (hatched bars) or uninfected reticuloctyes (clear bars). By day 23 more than 80 to 90% of the erythrocytes are uninfected normocytes. Parasitemia on day 3 is reduced compared to control animals which are absent in subsequent days due to control animal death. Quantification was performed on counting at least 500 erythrocytes per mouse and averaged in the groups of 4 or number of remaining mice. Counts are represented as number of infected or uninfected normocytes or reticulocytes out of 100. Error is standard deviation of means between up to 4 mice.

In the lethal *P. berghei*ANKA mouse model, FBS0701 showed a 50% reduction in day 5 parasitemia and delayed death by more than ten days at the 100 mg/kg dose, which was independent of single day dosing for one day, three days or seven days (not shown). The lethal *P. yoelii* model was used next because while lethal infection results from invasion of both normocytes and reticulocytes a recent vaccine study demonstrated that by largely restricting invasion to reticulocytes the infection was no longer lethal. [22]. We dosed mice with the FBS0701 by single oral dose either seven days or one day prior to parasite inoculation and also dosed a day after inoculation. All infected untreated control mice died by day 11. All the mice treated a day after infection with a single oral dose of the iron chelator survived (Figure 3). Surprisingly two to three of mice with FBS0701 pretreatment one or seven days prior to intraperitoneal *P. yoelii* infection still lived suggesting a lingering effect after the drug was cleared. In this model we again saw a small reduction in day 3 parasitemia. In all surviving mice, which had been dosed with FBS0701, the day 16 parasitemia was near 50% but confined to reticulocytes rather than normocytes (Figure 4). Resolution of parasitemia coincided with return of reticulocyte count to normal levels.

### Mouse Pharmacokinetics

**Figure 5:**
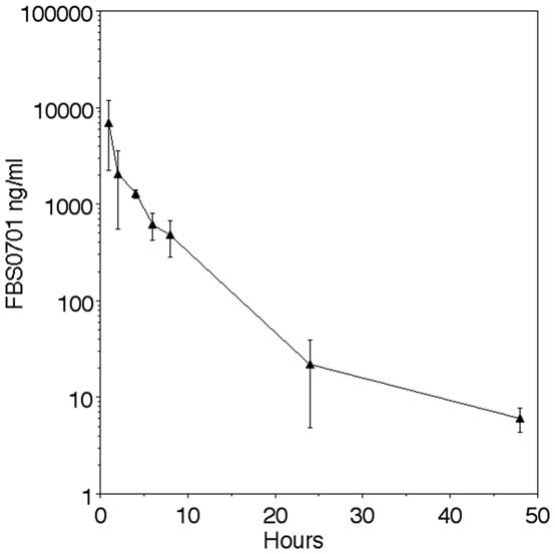
Pharmacokinetics of FBS0701 in mice show high peak values. 21 fed mice were given single oral dose at 100 mg/kg (filled triangle) and were sacrificed at indicated time with whole blood separated into erythrocytes and plasma. At least 200 microliters of plasma was analyzed for drug levels in the three mice at each time point. Error is standard deviation of the mean.

We performed a pharmacokinetic analysis of FBS0701 dosing in fed mice without malaria. 100 mg/kg dosing resulted in 17 µM levels at one hour that by 8 hours was close to 1 µM (Figure 5). These concentrations are below those easily achieved in humans [13].

### Removal of Labile Erythrocyte Iron

**Figure 6:**
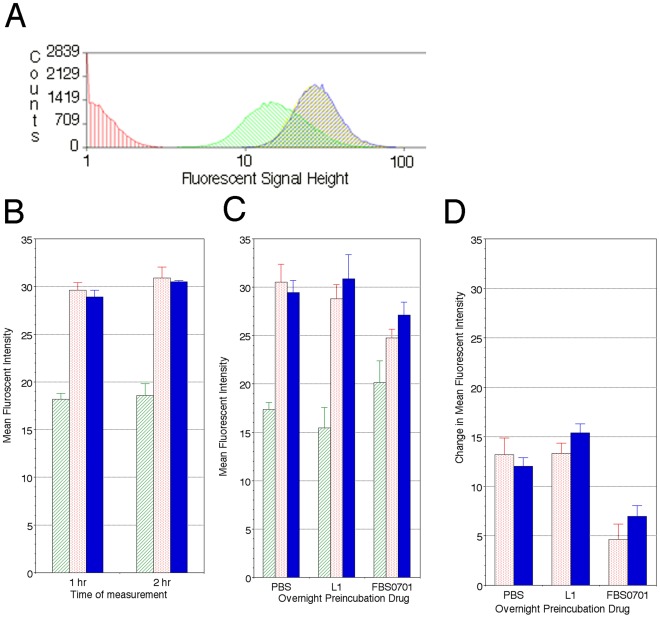
FBS0701 treatment chelates intracellular erythrocytic iron to the same extent as deferiprone but unlike deferiprone, removes iron from erythrocytes. Aliquots 1–5×10^6^ erythrocytes from two separate healthy donors were stained in the dark with 0.125 µM calcein-AM for 15 minutes at 37°C. Cells were washed twice with PBS and allowed to rest for 10 minutes at 37°C in the dark. Flow cytometry on FACS- Calibur records fluorescent signal height of calcein on 40,000 cells. A. Calcein-AM fluorescence histograms of unstained (no calcein) erythrocytes (red) and calcein-stained erythrocytes (green) followed by incubation without (green) or with iron chelator L1 (blue) or FBS0701(yellow) show an increase in fluorescent signal intensity with iron chelator indicating displacement of labile iron from calcein. The L1 and FBS0701 peaks overlap. B. Washed erythrocytes after calcein-AM staining were incubated with PBS (green hatched bars), 100 µM L1(-deferiprone) (red dotted bars) or 100 µM FBS0701 (blue solid bars) chelator for 1 hr or 2 hr. Both chelators were able to enter erythrocyte and chelate iron once bound to calcein to increase MFI. C. Erythrocytes were incubated with PBS, 100 µM L1 (deferiprone) or 100 µM FBS7010 chelator for 1 hr and washed twice with PBS and incubated overnight in PBS. Erythrocytes were then washed, stained with calcein-AM and incubated with either L1 (red dotted bars) or FBS0701 (blue solid bars). The baseline calcein fluorescence in FBS0701 pre-treated cells was higher indicating a lower concentration of labile iron in these erythrocytes, while baseline MFI in cells incubated with deferiprone have is essentially unchanged. D. Change in Mean Fluorescent Intensity is shown for each of the conditions described in C. In the FBS0701 pre-treated cells, the addition of 100 µM of either chelator resulted in a change in MFI of 5 compared to a change of 15 in cells treated with either PBS or deferiprone. The data in B was performed in three replicates on RBCs from a single blood donor while C and D was performed using RBCsfrom two different donors each in triplicate and mean and standard deviation (SD) of the 6 data points is shown. Student’s t-test shows a statistical significant difference in the change in MFI for both iron chelators and PBS in B and C. In C, baseline FBS0701 MFI is higher than both PBS and deferiprone (p<0.05)and in D the change in MFI with FBS0701 after the overnight pre-treatment is less than with PBS and deferiprone (student’s t-test, p<0.005).

Calcein has been used as an intracellular iron probe [23], [24]. Labile iron bound to low molecular weight, low affinity chelators binds and quenches calcein to generate the baseline equilibrium fluorescent intensity. The labile iron pools in normal erythrocytes has been determined to be approximately 1 µM [18], [19]. Similar to deferiprone, the iron chelator FBS0701 at 100 µM was able to enter erythrocytes as evidenced by the increase in fluorescent intensity consistent with the release of iron from calcein (Figure 6A and B). Incubation of the FBS0701 and deferiprone for either one or two hours showed no difference in endpoint fluorescence intensity. To measure the removal of intracellular iron by chelation, erythrocytes at 4×10^6^ cells/ml (0.125% hematocrit) were incubated for an hour with 100 µM of either deferiprone or FBS0701, washed and then placed in PBS overnight. Figure 6C shows that fluorescence in erythrocytes pre-treated with FBS0701 increased slightly from a mean fluorescence intensity (MFI) of 17 to 20. Pre-treatment with deferiprone showed a slight though statistically insignificant decrease in MFI. Addition of either iron chelators to cells previously incubated with FBS0701 showed a small rise of approximately 5 MFI compared to an increase of approximately 15 seen with either PBS or deferiprone-incubated cells demonstrating that in erythrocytes, FBS0701 depletes the labile pool of intracellular iron.

## Discussion

A bioavailable oral iron chelator is an attractive and proven objective as an antimalarial agent. Much of the lack of efficacy of previous iron chelators for malaria therapy can be attributed to pharmacokinetic limitations including bioavailability, poor iron clearance efficiency and short-half life. FBS0701 has high solubility and a *log p* of −1.22 permitting good oral bioavailability and much better iron clearance than deferrioxamine and deferipone. Here we demonstrate activity against blood stage *P. falciparum* and also single dose cure in a lethal mouse malaria model. A potential limitation is the known interference to the artemisinin class of drugs. However, the evidence from this work indicates that FBS0701 may remove an irreplaceable source of iron in normocytes in mice. Previous studies on where iron is removed with deferoxamine in the calcein measurements showed a decrease in labile iron in both infected and uninfected erythrocytes. We have not demonstrated a decrease in nonheme iron in these studies but suggest that the effect we see in vivo is not from a new recompartmentalization of iron but from a decrease in erythrocyte levels. Consistent with other reports [9], [16], [21], we have demonstrated *in vitro* interference when both iron chelators and artemisinin are present simultaneously, but have not tested whether pre-treatment with FBS0701 before infection interferes with artemisinin activity.

Interestingly, the dose of iron chelator largely restricted lethal *P. yoelii* to young erythrocytes even almost 28 days after the drug was dosed and three weeks into infection. Mouse erythrocytes have a life span of about 60 days [25], [26]. Additional studies of the timing of iron chelator days to weeks before lethal *P. yoelii* infection will provide evidence to replenishment of this erythrocyte bioavailable source of iron for *Plasmodium*.

The mouse pharmacokinetic parameters are close to those obtained in the rat model [12]. Most of the iron-FBS0701 complex was excreted in the bile while free FBS0701 has renal excretion [12]. The relative level of FBS0701 in the rodent shows liver>kidney>plasma>pancreas>heart. Interestingly, humans show approximately ten times higher plasma concentrations compared to mouse for a given mg/kg dose. However, in studies comparing dose proportionality for cancer drugs where toxicity is a larger issue 100 mg/kg dose in mice represents a dose of 300 mg/m^2^ while a 10 mg/kg in humans is equivalent to 370 mg/m^2^ [27]. The implications are that in humans effective drug concentrations above minimal *P. falciparum* inhibition concentrations last less than ten hours. In the *P. falciparum in vitro* drug testing the drug is at continuous concentration for three days. In the mouse *in vivo* studies here we have demonstrated a persistent effect weeks after the drug was dosed. This data indicate that iron chelation is able to remove a bioavailable source which persists and plays a role in parasite inhibition. Unlike many of the long half-life blood stage active quinolines which rely on time above inhibition concentration to kill parasites, FBS0701 has an effect possibly by perturbing an iron compartment in the circulating erythrocytes.

The determination of relative labile iron concentrations using calcein indicates FBS0701 is able enter erythrocytes and bind iron bound to calcein. More importantly, in contrast to deferiprone, FBS0701 was able to remove labile iron from erythrocytes, an effect that persisted for at least 16 hours. This is the first study to demonstrate the egress of labile iron complexed with a chelator complex from erythrocytes. This has important clinical implications for the use of an iron chelator in the treatment of malaria as well as the ability of FBS0701 to remove iron from other cell types.

In the malaria iron chelator literature there is some debate whether iron chelators inhibit by limiting DNA replication which in many bacteria and mammalian cells is cytostatic or whether iron chelators are toxic and therefore parasiticidal to *Plasmodium*. Early stage gametocytes do not replicate DNA, so the data which show early stage inhibition with FBS0701 may indicate an additional toxic mechanism of inhibition at the high dose used in the gametocyte assay at the stages digesting the iron and hemoglobin rich erythrocyte cytoplasm.

In summary, FBS0701 demonstrates more potent inhibition for *P. falciparum* than previous iron chelators in clinical use. The activity persists in mice after the drug is below inhibition concentrations of approximately 5 uM. Our gametocyte data suggest a combined toxic mechanism in presence of erythrocyte cytosol ingestion in addition to the proposed mechanism of limiting DNA synthesis. In the lethal *P. yoelii* murine malaria model we show single oral dose cure. The removal of erythrocytic iron from intracellular bioavailable pools likely contributes to both the antimalarial activity and duration of effect of FBS0701 accounting for these effects in the relative absence of drug in plasma. FBS0701 may find clinically utility as monotherapy, a malarial prophylactic or, more likely, in combination with other antimalarials after further preclinical testing to investigate liver stage activity, duration of blood stage interference with the artemisinins or possibly inhibition of transmission in mosquito stages ingesting FBS0701 exposed gametocytes.
